# {1,1′-[(2,2-Dimethyl­propane-1,3-di­yl)bis­(nitrilo­methyl­idyne)]di-2-naphthol­ato}dioxidomolybdenum(VI) dichloro­methane 1.75-solvate

**DOI:** 10.1107/S1600536809032759

**Published:** 2009-08-22

**Authors:** Niaz Monadi, Iran Sheikhshoaie, Abdoreza Rezaeifard, Helen Stoeckli-Evans

**Affiliations:** a Chemistry Department, Shahid Bahonar University of Kerman, Kerman, Iran; b Chemistry Department, Birjand University, Birjand, Iran; c Institute of Physics, University of Neuchâtel, Rue Emile-Argand 11, CH-2009 Neuchâtel, Switzerland

## Abstract

In the crystal structure of the title compound, [Mo(C_27_H_24_N_2_O_2_)O_2_]·1.75CH_2_Cl_2_, the Mo^VI^ ion is coordinated by two oxide O atoms and by two O and two N atoms of the tetra­dentate 1,1′-[(2,2-dimethyl­propane-1,3-di­yl)bis­(nitrilo­methyl­idyne)]di-2-naphtholate Schiff base ligand in a distorted octa­hedral configuration. The compound crystallizes with 1.75 mol­ecules of dichloro­methane per complex mol­ecule. In the crystal, symmetry-related mol­ecules are linked by a number of C—H⋯O inter­actions involving both the Schiff base ligand and the partly disordered dichloro­methane solvent mol­ecules, leading to the formation of a two-dimensional network extending parallel to (101).

## Related literature

For the chemistry of molybdenum(VI)–Schiff base complexes and related structures with O=Mo=O units (metal oxidation state +VI), see: Abbasi *et al.* (2008 ▶); Arnaiz *et al.* (2000 ▶); Holm *et al.* (1996 ▶); Maurya *et al.* (1997 ▶); Nakayima *et al.* (1998 ▶); Rao *et al.* (1998 ▶); Sheikhshoaie *et al.* (2009 ▶); Syamal & Maurya (1989 ▶).
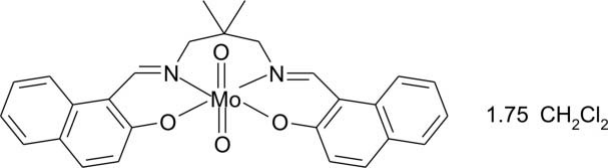


## Experimental

### 

#### Crystal data

[Mo(C_27_H_24_N_2_O_2_)O_2_]·1.75CH_2_Cl_2_
*M* *_r_* = 685.04
Monoclinic, 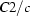

*a* = 27.6049 (18) Å
*b* = 10.7743 (8) Å
*c* = 21.6474 (14) Å
β = 112.861 (7)°
*V* = 5932.7 (7) Å^3^
*Z* = 8
Mo *K*α radiation
μ = 0.79 mm^−1^
*T* = 173 K
0.38 × 0.23 × 0.18 mm

#### Data collection

Stoe IPDS diffractometer
Absorption correction: multi-scan (*MULscanABS* in *PLATON*; Spek, 2009 ▶) *T*_min_ = 0.833, *T*_max_ = 0.864
21863 measured reflections
5537 independent reflections
4789 reflections with *I* > 2σ(*I*)
*R*_int_ = 0.024

#### Refinement

*R*[*F* ^2^ > 2σ(*F* ^2^)] = 0.035
*wR*(*F* ^2^) = 0.106
*S* = 1.12
5537 reflections
372 parameters
H-atom parameters constrained
Δρ_max_ = 1.08 e Å^−3^
Δρ_min_ = −0.83 e Å^−3^

### 

Data collection: *EXPOSE* in *IPDS-I* (Stoe & Cie, 2000 ▶); cell refinement: *CELL* in *IPDS-I* (Stoe & Cie, 2000 ▶); data reduction: *INTEGRATE* in *IPDS-I* (Stoe & Cie, 2000 ▶); program(s) used to solve structure: *SHELXS97* (Sheldrick, 2008 ▶); program(s) used to refine structure: *SHELXL97* (Sheldrick, 2008 ▶); molecular graphics: *PLATON* (Spek, 2009 ▶) and *Mercury* (Macrae *et al.*, 2006 ▶); software used to prepare material for publication: *SHELXL97*.

## Supplementary Material

Crystal structure: contains datablocks I, global. DOI: 10.1107/S1600536809032759/wm2248sup1.cif

Structure factors: contains datablocks I. DOI: 10.1107/S1600536809032759/wm2248Isup2.hkl

Additional supplementary materials:  crystallographic information; 3D view; checkCIF report

## Figures and Tables

**Table 1:** Selected bond lengths (Å)

Mo1—O1	2.0888 (19)
Mo1—O2	1.957 (2)
Mo1—O3	1.702 (2)
Mo1—O4	1.713 (2)
Mo1—N1	2.118 (2)
Mo1—N2	2.297 (2)

**Table 2:** Hydrogen-bond geometry (Å, °)

*D*—H⋯*A*	*D*—H	H⋯*A*	*D*⋯*A*	*D*—H⋯*A*
C6—H6⋯O3^i^	0.95	2.43	3.328 (5)	159
C11—H11⋯O4^ii^	0.95	2.38	3.297 (3)	161
C12—H12*A*⋯O4	0.99	2.47	2.974 (4)	111
C27—H27*A*⋯O3^ii^	0.98	2.55	3.490 (4)	161
C28—H28*A*⋯O2^iii^	0.99	2.40	3.213 (5)	139
C28—H28*A*⋯O4^iii^	0.99	2.53	3.429 (6)	151

Symmetry codes: (i) 
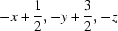
; (ii) 
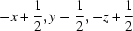
; (iii) 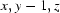.

**Fig. 1.**
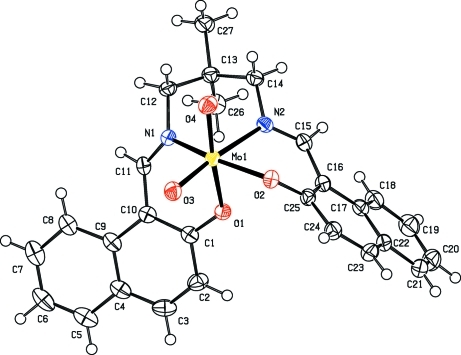
A view of the molecular structure of the title complex, with the thermal ellipsoids drawn at the 50% probability level. The dichloromethane molecules of crystallization have been omitted for clarity.

**Fig. 2.**
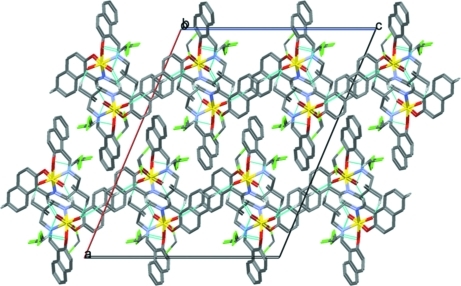
A view along the b axis of the crystal packing of the title complex. The H-atoms not involved in C—H···O interactions (dotted blue lines) were omitted for clarity.

## supplementary crystallographic information

### Comment

Numerous molybdenum(VI) Schiff base complexes have been extensively investigated for over the past twenty years, due to their importance in the domains of stereochemistry (Maurya *et al.*, 1997), structural chemistry (Nakayima, *et al.*, 1998; Syamal & Maurya, 1989), analytical chemistry (Rao *et al.*, 1998), bioinorganic chemistry (Holm *et al.*, 1996), and oxidation catalysis (Abbasi *et al.*, 2008; Arnaiz *et al.*, 2000; Sheikhshoaie *et al.*, 2009). Continuing our interest in the structural chemistry of dioxidomolybdenum(VI) Schiff base complexes, we have synthesized and structurally characterized the title compound.

The molecular structure of the title complex is illustrated in Fig. 1, and geometrical parameters are available in the archived CIF. The Mo^VI^ atom is in a distorted octahedral environment being coordinated by two oxido O atoms (O1 and O2) and four atoms (two oxygen and two nitrogen atoms) of the tetradentate Schiff base ligand 1,1'-[(2,2-dimethylpropane-1,3-diyl)bis(nitrilomethylidyne)]di-2-naphtholate. The Mo—O distances of the oxido ligands (Mo1O3 and Mo1O4) are significantly shorter [1.702 (2) and 1.713 (2) Å, respectively] than the corresponding Mo—O distances to the O-atoms (O2 and O1) of the tetradentate Schiff base ligand, 1.957 (2) and 2.0888 (19) Å, respectively. The Mo—N distances, to atoms N1 and N2, are even longer being 2.118 (2) and 2.297 (2) Å, respectively.

In the crystal, molecules are linked by a number of C—H···O interactions, involving both the Schiff base ligand and the solvent molecules of crystallization, leading to the formation of a two-dimensional network extending in the (101) plane (Table 1 and Fig. 2).

### Experimental

The title dioxidomolybdenum (VI) complex was prepared by mixing MoO_2_(acac)_2_ with the ligand, 2,2'-[(2,2-dimethylpropane-1,3-diyl)bis(nitrilomethylidyne)]-dinaphtholate, in a 1:1 molar ratio using 25 ml of dry methanol as solvent, followed by refluxing the solution for 3 h. The small reddish crystals that formed were filtered off and recrystallized from dichloromethane.

### Refinement

All H-atoms were placed in the calculated positions and treated as riding atoms: C—H = 0.95 - 0.99 Å with *U*_iso_(H) = k × *U*_eq_(parent C-atom), where k = 1.2 for aromatic H-atoms, and 1.5 for methyl H-atoms. The compound crystallizes with 1.75 molecules of dichloromethane per molecule of complex. In one of these molecules a chlorine atom (Cl1A/Cl1B) is positionally disordered with occupancies of 0.75/0.25. Using the one-circle Stoe Image Plate Diffraction System it is not possible to measure 100% of the Ewald sphere, and here only 95% was accessible.

### Crystal data

**Table:**

[Mo(C_27_H_24_N_2_O_2_)O_2_]·1.75CH_2_Cl_2_	*F*(000) = 2780
*M**_r_* = 685.04	*D*_x_ = 1.534 Mg m^−^^3^
Monoclinic, *C*2/*c*	Mo *K*α radiation, λ = 0.71073 Å
Hall symbol: -C 2yc	Cell parameters from 7998 reflections
*a* = 27.6049 (18) Å	θ = 2.0–26.1°
*b* = 10.7743 (8) Å	µ = 0.79 mm^−^^1^
*c* = 21.6474 (14) Å	*T* = 173 K
β = 112.861 (7)°	Block, red
*V* = 5932.7 (7) Å^3^	0.38 × 0.23 × 0.18 mm
*Z* = 8	

### Data collection

**Table:**

Stoe IPDS diffractometer	5537 independent reflections
Radiation source: fine-focus sealed tube	4789 reflections with *I* > 2σ(*I*)
graphite	*R*_int_ = 0.024
φ rotation scans	θ_max_ = 26.0°, θ_min_ = 2.2°
Absorption correction: multi-scan (MULscanABS in *PLATON*; Spek, 2009)	*h* = −33→33
*T*_min_ = 0.833, *T*_max_ = 0.864	*k* = −13→13
21863 measured reflections	*l* = −26→26

### Refinement

**Table:**

Refinement on *F*^2^	Primary atom site location: structure-invariant direct methods
Least-squares matrix: full	Secondary atom site location: difference Fourier map
*R*[*F*^2^ > 2σ(*F*^2^)] = 0.035	Hydrogen site location: inferred from neighbouring sites
*wR*(*F*^2^) = 0.106	H-atom parameters constrained
*S* = 1.12	*w* = 1/[σ^2^(*F*_o_^2^) + (0.0575*P*)^2^ + 13.6226*P*] where *P* = (*F*_o_^2^ + 2*F*_c_^2^)/3
5537 reflections	(Δ/σ)_max_ = 0.001
372 parameters	Δρ_max_ = 1.08 e Å^−^^3^
0 restraints	Δρ_min_ = −0.83 e Å^−^^3^

### Special details

**Table:**

Geometry. Bond distances, angles *etc*. have been calculated using the rounded fractional coordinates. All su's are estimated from the variances of the (full) variance-covariance matrix. The cell e.s.d.'s are taken into account in the estimation of distances, angles and torsion angles

### Fractional atomic coordinates and isotropic or equivalent isotropic displacement parameters (Å^2^)

**Table:**

	*x*	*y*	*z*	*U*_iso_*/*U*_eq_	Occ. (<1)
Mo1	0.15195 (1)	0.99201 (2)	0.16605 (1)	0.0203 (1)	
O1	0.11880 (7)	0.83854 (17)	0.10537 (10)	0.0244 (5)	
O2	0.07965 (7)	1.05359 (17)	0.13420 (10)	0.0243 (5)	
O3	0.17351 (8)	1.04666 (19)	0.10747 (10)	0.0294 (6)	
O4	0.18137 (8)	1.08694 (18)	0.23387 (10)	0.0292 (6)	
N1	0.21210 (9)	0.8581 (2)	0.20744 (11)	0.0230 (7)	
N2	0.11991 (9)	0.8840 (2)	0.23353 (11)	0.0226 (6)	
C1	0.14254 (11)	0.7716 (2)	0.07556 (14)	0.0240 (8)	
C2	0.11155 (12)	0.7170 (3)	0.01261 (15)	0.0320 (9)	
C3	0.13435 (13)	0.6494 (3)	−0.02156 (16)	0.0351 (9)	
C4	0.18908 (13)	0.6260 (3)	0.00395 (15)	0.0297 (9)	
C5	0.21229 (15)	0.5592 (3)	−0.03334 (16)	0.0359 (10)	
C6	0.26506 (15)	0.5380 (3)	−0.00885 (17)	0.0378 (10)	
C7	0.29652 (14)	0.5838 (3)	0.05388 (18)	0.0373 (10)	
C8	0.27554 (13)	0.6506 (3)	0.09176 (16)	0.0312 (9)	
C9	0.22085 (12)	0.6729 (2)	0.06788 (14)	0.0258 (8)	
C10	0.19693 (11)	0.7471 (2)	0.10402 (14)	0.0240 (8)	
C11	0.22523 (11)	0.7765 (2)	0.17248 (14)	0.0236 (8)	
C12	0.23835 (11)	0.8489 (3)	0.28089 (14)	0.0259 (8)	
C13	0.20409 (11)	0.7852 (3)	0.31281 (14)	0.0264 (8)	
C14	0.15487 (11)	0.8626 (3)	0.30383 (14)	0.0265 (8)	
C15	0.07193 (11)	0.8512 (2)	0.21471 (14)	0.0243 (8)	
C16	0.03019 (11)	0.8852 (3)	0.15189 (14)	0.0237 (8)	
C17	−0.01851 (11)	0.8180 (3)	0.12751 (14)	0.0258 (8)	
C18	−0.02482 (13)	0.7013 (3)	0.15323 (16)	0.0317 (9)	
C19	−0.07116 (14)	0.6372 (3)	0.12614 (18)	0.0396 (11)	
C20	−0.11356 (13)	0.6855 (3)	0.07190 (19)	0.0412 (11)	
C21	−0.10943 (12)	0.7981 (3)	0.04577 (16)	0.0347 (9)	
C22	−0.06213 (11)	0.8663 (3)	0.07250 (15)	0.0277 (8)	
C23	−0.05656 (12)	0.9810 (3)	0.04395 (16)	0.0311 (9)	
C24	−0.00985 (11)	1.0411 (3)	0.06555 (15)	0.0276 (8)	
C25	0.03480 (11)	0.9914 (2)	0.11817 (15)	0.0231 (8)	
C26	0.18840 (13)	0.6549 (3)	0.28459 (17)	0.0347 (9)	
C27	0.23717 (13)	0.7789 (3)	0.38843 (15)	0.0342 (9)	
Cl1A	0.11134 (12)	0.36651 (18)	0.28031 (10)	0.1016 (8)	0.800
Cl2	0.06716 (6)	0.14580 (16)	0.31642 (8)	0.0913 (6)	
C28	0.0768 (2)	0.2286 (5)	0.2529 (2)	0.0657 (17)	
Cl1B	0.0605 (5)	0.3839 (7)	0.2649 (4)	0.102 (4)	0.200
Cl3	0.02059 (6)	0.4230 (2)	0.09019 (10)	0.0919 (8)	0.750
Cl4	0.13068 (6)	0.4167 (3)	0.11352 (11)	0.0993 (8)	0.750
C29	0.0693 (3)	0.3744 (8)	0.0643 (4)	0.088 (3)	0.750
H2	0.07450	0.72800	−0.00560	0.0380*	
H3	0.11280	0.61660	−0.06420	0.0420*	
H5	0.19070	0.52830	−0.07640	0.0430*	
H6	0.28020	0.49240	−0.03440	0.0450*	
H7	0.33330	0.56880	0.07100	0.0450*	
H8	0.29800	0.68190	0.13430	0.0370*	
H11	0.25700	0.73220	0.19510	0.0280*	
H12A	0.24750	0.93330	0.29990	0.0310*	
H12B	0.27150	0.80150	0.29240	0.0310*	
H14A	0.13480	0.81950	0.32670	0.0320*	
H14B	0.16620	0.94380	0.32610	0.0320*	
H15	0.06280	0.80030	0.24440	0.0290*	
H18	0.00360	0.66670	0.19000	0.0380*	
H19	−0.07450	0.55900	0.14440	0.0480*	
H20	−0.14540	0.63980	0.05320	0.0490*	
H21	−0.13860	0.83090	0.00930	0.0420*	
H23	−0.08610	1.01640	0.00910	0.0370*	
H24	−0.00690	1.11740	0.04530	0.0330*	
H26A	0.22010	0.60530	0.29290	0.0520*	
H26B	0.16780	0.65990	0.23620	0.0520*	
H26C	0.16720	0.61560	0.30650	0.0520*	
H27A	0.26850	0.72800	0.39650	0.0510*	
H27B	0.24790	0.86290	0.40570	0.0510*	
H27C	0.21620	0.74190	0.41130	0.0510*	
H28A	0.09620	0.17560	0.23290	0.0790*	0.800
H28B	0.04210	0.24780	0.21730	0.0790*	0.800
H28C	0.11240	0.21750	0.25530	0.0790*	0.200
H28D	0.05230	0.20120	0.20920	0.0790*	0.200
H29A	0.06810	0.28270	0.06100	0.1060*	0.750
H29B	0.06140	0.40740	0.01870	0.1060*	0.750

### Atomic displacement parameters (Å^2^)

**Table:**

	*U*^11^	*U*^22^	*U*^33^	*U*^12^	*U*^13^	*U*^23^
Mo1	0.0189 (1)	0.0204 (1)	0.0216 (1)	−0.0010 (1)	0.0080 (1)	−0.0015 (1)
O1	0.0227 (9)	0.0256 (9)	0.0249 (10)	−0.0011 (7)	0.0092 (8)	−0.0046 (7)
O2	0.0215 (9)	0.0231 (9)	0.0287 (10)	0.0001 (7)	0.0103 (8)	0.0010 (8)
O3	0.0272 (10)	0.0320 (11)	0.0308 (11)	−0.0018 (8)	0.0133 (9)	0.0012 (8)
O4	0.0278 (10)	0.0257 (10)	0.0315 (11)	−0.0013 (8)	0.0086 (9)	−0.0053 (8)
N1	0.0193 (11)	0.0271 (12)	0.0220 (11)	0.0005 (9)	0.0074 (9)	−0.0015 (9)
N2	0.0246 (11)	0.0235 (11)	0.0205 (11)	0.0036 (9)	0.0097 (10)	0.0005 (9)
C1	0.0292 (14)	0.0198 (12)	0.0249 (13)	−0.0016 (10)	0.0127 (12)	0.0000 (10)
C2	0.0312 (15)	0.0290 (15)	0.0301 (15)	0.0010 (12)	0.0058 (13)	−0.0057 (12)
C3	0.0465 (18)	0.0275 (15)	0.0261 (15)	−0.0007 (13)	0.0086 (14)	−0.0046 (11)
C4	0.0446 (17)	0.0215 (13)	0.0280 (14)	−0.0004 (12)	0.0196 (14)	0.0005 (11)
C5	0.060 (2)	0.0229 (14)	0.0318 (16)	−0.0016 (13)	0.0254 (16)	−0.0004 (12)
C6	0.062 (2)	0.0243 (14)	0.0428 (18)	0.0024 (14)	0.0375 (18)	0.0004 (13)
C7	0.0467 (19)	0.0271 (15)	0.0501 (19)	0.0051 (13)	0.0318 (17)	0.0059 (13)
C8	0.0372 (16)	0.0276 (14)	0.0352 (16)	0.0024 (12)	0.0212 (14)	0.0019 (12)
C9	0.0358 (15)	0.0181 (12)	0.0290 (14)	0.0015 (11)	0.0186 (13)	0.0029 (10)
C10	0.0277 (14)	0.0205 (12)	0.0243 (13)	−0.0003 (10)	0.0107 (12)	0.0001 (10)
C11	0.0215 (13)	0.0247 (13)	0.0278 (14)	0.0021 (10)	0.0132 (12)	0.0015 (10)
C12	0.0224 (13)	0.0310 (14)	0.0203 (13)	0.0046 (11)	0.0038 (11)	−0.0023 (11)
C13	0.0263 (14)	0.0291 (14)	0.0221 (13)	0.0048 (11)	0.0077 (12)	−0.0005 (11)
C14	0.0271 (14)	0.0318 (14)	0.0206 (13)	0.0033 (11)	0.0094 (12)	0.0012 (11)
C15	0.0267 (14)	0.0241 (13)	0.0257 (14)	0.0008 (10)	0.0141 (12)	0.0019 (10)
C16	0.0222 (13)	0.0257 (13)	0.0243 (13)	0.0024 (10)	0.0104 (11)	−0.0016 (10)
C17	0.0267 (14)	0.0285 (14)	0.0252 (14)	−0.0004 (11)	0.0133 (12)	−0.0051 (11)
C18	0.0350 (16)	0.0318 (15)	0.0327 (16)	−0.0036 (12)	0.0180 (14)	−0.0024 (12)
C19	0.0454 (19)	0.0353 (17)	0.0453 (19)	−0.0133 (14)	0.0254 (17)	−0.0073 (14)
C20	0.0330 (17)	0.0463 (19)	0.0472 (19)	−0.0173 (14)	0.0188 (16)	−0.0143 (15)
C21	0.0243 (14)	0.0477 (18)	0.0331 (16)	−0.0042 (13)	0.0121 (13)	−0.0106 (14)
C22	0.0216 (13)	0.0359 (15)	0.0274 (14)	−0.0016 (11)	0.0116 (12)	−0.0072 (12)
C23	0.0238 (14)	0.0383 (16)	0.0279 (15)	0.0042 (12)	0.0065 (13)	−0.0009 (12)
C24	0.0261 (14)	0.0284 (14)	0.0283 (14)	0.0043 (11)	0.0106 (12)	0.0036 (11)
C25	0.0209 (13)	0.0230 (13)	0.0263 (14)	0.0011 (10)	0.0101 (12)	−0.0035 (10)
C26	0.0378 (17)	0.0294 (15)	0.0369 (17)	0.0023 (13)	0.0145 (14)	0.0008 (12)
C27	0.0332 (16)	0.0433 (18)	0.0248 (15)	0.0124 (13)	0.0097 (13)	0.0043 (12)
Cl1A	0.150 (2)	0.0683 (11)	0.0748 (11)	−0.0216 (12)	0.0309 (13)	−0.0188 (9)
Cl2	0.0686 (8)	0.1124 (12)	0.0813 (9)	0.0082 (8)	0.0163 (7)	0.0270 (8)
C28	0.067 (3)	0.073 (3)	0.064 (3)	−0.009 (2)	0.033 (2)	−0.027 (2)
Cl1B	0.166 (9)	0.060 (4)	0.073 (4)	0.011 (5)	0.040 (5)	−0.022 (3)
Cl3	0.0444 (8)	0.150 (2)	0.0799 (12)	0.0113 (10)	0.0225 (8)	−0.0222 (12)
Cl4	0.0402 (8)	0.157 (2)	0.0875 (13)	0.0037 (10)	0.0105 (8)	0.0133 (13)
C29	0.057 (4)	0.114 (6)	0.100 (6)	−0.009 (4)	0.038 (4)	−0.048 (5)

### Geometric parameters (Å, °)

**Table:**

Mo1—O1	2.0888 (19)	C18—C19	1.369 (5)
Mo1—O2	1.957 (2)	C19—C20	1.397 (5)
Mo1—O3	1.702 (2)	C20—C21	1.362 (5)
Mo1—O4	1.713 (2)	C21—C22	1.412 (5)
Mo1—N1	2.118 (2)	C22—C23	1.417 (5)
Mo1—N2	2.297 (2)	C23—C24	1.354 (5)
Cl1A—C28	1.741 (6)	C24—C25	1.419 (4)
Cl1B—C28	1.778 (10)	C2—H2	0.9500
Cl2—C28	1.744 (5)	C3—H3	0.9500
Cl3—C29	1.727 (9)	C5—H5	0.9500
Cl4—C29	1.677 (9)	C6—H6	0.9500
O1—C1	1.302 (3)	C7—H7	0.9500
O2—C25	1.330 (4)	C8—H8	0.9500
N1—C12	1.472 (4)	C11—H11	0.9500
N1—C11	1.300 (4)	C12—H12A	0.9900
N2—C14	1.470 (4)	C12—H12B	0.9900
N2—C15	1.275 (4)	C14—H14B	0.9900
C1—C10	1.409 (4)	C14—H14A	0.9900
C1—C2	1.425 (4)	C15—H15	0.9500
C2—C3	1.355 (5)	C18—H18	0.9500
C3—C4	1.415 (5)	C19—H19	0.9500
C4—C5	1.408 (5)	C20—H20	0.9500
C4—C9	1.413 (4)	C21—H21	0.9500
C5—C6	1.362 (6)	C23—H23	0.9500
C6—C7	1.388 (5)	C24—H24	0.9500
C7—C8	1.377 (5)	C26—H26C	0.9800
C8—C9	1.413 (5)	C26—H26B	0.9800
C9—C10	1.445 (4)	C26—H26A	0.9800
C10—C11	1.417 (4)	C27—H27A	0.9800
C12—C13	1.533 (4)	C27—H27B	0.9800
C13—C26	1.525 (5)	C27—H27C	0.9800
C13—C27	1.535 (4)	C28—H28A	0.9900
C13—C14	1.541 (5)	C28—H28D	0.9700
C15—C16	1.447 (4)	C28—H28B	0.9900
C16—C25	1.389 (4)	C28—H28C	0.9700
C16—C17	1.435 (5)	C29—H29A	0.9900
C17—C18	1.413 (5)	C29—H29B	0.9900
C17—C22	1.423 (4)		
			
O1—Mo1—O2	84.79 (8)	C1—C2—H2	120.00
O1—Mo1—O3	90.00 (9)	C3—C2—H2	120.00
O1—Mo1—O4	163.25 (9)	C2—C3—H3	119.00
O1—Mo1—N1	78.85 (8)	C4—C3—H3	119.00
O1—Mo1—N2	79.39 (8)	C4—C5—H5	119.00
O2—Mo1—O3	102.96 (10)	C6—C5—H5	119.00
O2—Mo1—O4	100.28 (10)	C5—C6—H6	120.00
O2—Mo1—N1	154.77 (9)	C7—C6—H6	120.00
O2—Mo1—N2	78.68 (9)	C6—C7—H7	119.00
O3—Mo1—O4	104.21 (10)	C8—C7—H7	119.00
O3—Mo1—N1	96.16 (10)	C7—C8—H8	120.00
O3—Mo1—N2	169.12 (9)	C9—C8—H8	120.00
O4—Mo1—N1	90.65 (9)	N1—C11—H11	117.00
O4—Mo1—N2	85.92 (9)	C10—C11—H11	117.00
N1—Mo1—N2	79.52 (9)	N1—C12—H12A	109.00
Mo1—O1—C1	124.64 (18)	N1—C12—H12B	109.00
Mo1—O2—C25	129.78 (16)	C13—C12—H12A	109.00
Mo1—N1—C11	124.36 (19)	C13—C12—H12B	109.00
Mo1—N1—C12	118.61 (18)	H12A—C12—H12B	108.00
C11—N1—C12	116.8 (2)	N2—C14—H14A	109.00
Mo1—N2—C14	118.55 (19)	N2—C14—H14B	109.00
Mo1—N2—C15	123.10 (18)	C13—C14—H14A	109.00
C14—N2—C15	118.0 (2)	C13—C14—H14B	109.00
O1—C1—C2	118.2 (3)	H14A—C14—H14B	108.00
O1—C1—C10	122.9 (2)	N2—C15—H15	117.00
C2—C1—C10	118.9 (3)	C16—C15—H15	117.00
C1—C2—C3	120.7 (3)	C17—C18—H18	119.00
C2—C3—C4	122.3 (3)	C19—C18—H18	119.00
C3—C4—C5	121.5 (3)	C18—C19—H19	120.00
C3—C4—C9	118.7 (3)	C20—C19—H19	120.00
C5—C4—C9	119.8 (3)	C19—C20—H20	120.00
C4—C5—C6	121.3 (3)	C21—C20—H20	120.00
C5—C6—C7	119.3 (3)	C20—C21—H21	120.00
C6—C7—C8	121.4 (4)	C22—C21—H21	120.00
C7—C8—C9	120.5 (3)	C22—C23—H23	119.00
C4—C9—C8	117.8 (3)	C24—C23—H23	119.00
C4—C9—C10	119.2 (3)	C23—C24—H24	120.00
C8—C9—C10	122.9 (3)	C25—C24—H24	120.00
C1—C10—C9	120.0 (3)	C13—C26—H26A	110.00
C1—C10—C11	118.5 (3)	C13—C26—H26B	110.00
C9—C10—C11	120.5 (3)	C13—C26—H26C	109.00
N1—C11—C10	126.5 (3)	H26A—C26—H26B	109.00
N1—C12—C13	112.7 (2)	H26A—C26—H26C	109.00
C12—C13—C14	111.7 (3)	H26B—C26—H26C	109.00
C12—C13—C26	111.1 (3)	C13—C27—H27A	109.00
C12—C13—C27	106.6 (3)	C13—C27—H27B	109.00
C14—C13—C26	110.4 (3)	C13—C27—H27C	109.00
C14—C13—C27	106.8 (2)	H27A—C27—H27B	109.00
C26—C13—C27	110.2 (3)	H27A—C27—H27C	110.00
N2—C14—C13	114.1 (2)	H27B—C27—H27C	109.00
N2—C15—C16	125.4 (3)	Cl1A—C28—Cl2	113.5 (2)
C15—C16—C17	120.5 (3)	Cl1B—C28—Cl2	104.0 (4)
C15—C16—C25	120.0 (3)	Cl1A—C28—H28A	109.00
C17—C16—C25	119.1 (3)	Cl1A—C28—H28B	109.00
C16—C17—C18	123.1 (3)	Cl2—C28—H28A	109.00
C16—C17—C22	119.4 (3)	Cl2—C28—H28B	109.00
C18—C17—C22	117.5 (3)	Cl2—C28—H28C	111.00
C17—C18—C19	121.2 (3)	Cl2—C28—H28D	111.00
C18—C19—C20	120.7 (3)	Cl1B—C28—H28C	115.00
C19—C20—C21	120.2 (3)	Cl1B—C28—H28D	108.00
C20—C21—C22	120.5 (3)	H28A—C28—H28B	108.00
C17—C22—C21	120.0 (3)	H28C—C28—H28D	109.00
C17—C22—C23	119.0 (3)	Cl3—C29—Cl4	115.7 (5)
C21—C22—C23	121.0 (3)	Cl3—C29—H29A	109.00
C22—C23—C24	121.1 (3)	Cl3—C29—H29B	108.00
C23—C24—C25	120.7 (3)	Cl4—C29—H29A	108.00
O2—C25—C16	123.0 (3)	Cl4—C29—H29B	108.00
O2—C25—C24	116.7 (2)	H29A—C29—H29B	107.00
C16—C25—C24	120.3 (3)		
			
O2—Mo1—O1—C1	149.5 (2)	C9—C4—C5—C6	0.4 (5)
O3—Mo1—O1—C1	46.5 (2)	C3—C4—C9—C8	−178.7 (3)
N1—Mo1—O1—C1	−49.8 (2)	C3—C4—C9—C10	−2.4 (4)
N2—Mo1—O1—C1	−131.1 (2)	C5—C4—C9—C8	0.3 (4)
O1—Mo1—O2—C25	33.1 (2)	C5—C4—C9—C10	176.6 (3)
O3—Mo1—O2—C25	121.9 (2)	C4—C5—C6—C7	−0.4 (5)
O4—Mo1—O2—C25	−130.7 (2)	C5—C6—C7—C8	−0.3 (5)
N1—Mo1—O2—C25	−16.4 (4)	C6—C7—C8—C9	0.9 (5)
N2—Mo1—O2—C25	−47.1 (2)	C7—C8—C9—C4	−0.9 (4)
O1—Mo1—N1—C11	37.9 (2)	C7—C8—C9—C10	−177.1 (3)
O1—Mo1—N1—C12	−137.0 (2)	C4—C9—C10—C1	0.2 (4)
O2—Mo1—N1—C11	88.4 (3)	C4—C9—C10—C11	168.5 (3)
O2—Mo1—N1—C12	−86.5 (3)	C8—C9—C10—C1	176.3 (3)
O3—Mo1—N1—C11	−50.9 (2)	C8—C9—C10—C11	−15.4 (4)
O3—Mo1—N1—C12	134.2 (2)	C1—C10—C11—N1	−23.9 (4)
O4—Mo1—N1—C11	−155.3 (2)	C9—C10—C11—N1	167.7 (3)
O4—Mo1—N1—C12	29.9 (2)	N1—C12—C13—C14	−65.6 (3)
N2—Mo1—N1—C11	119.0 (2)	N1—C12—C13—C26	58.2 (3)
N2—Mo1—N1—C12	−55.9 (2)	N1—C12—C13—C27	178.2 (2)
O1—Mo1—N2—C14	131.0 (2)	C12—C13—C14—N2	60.7 (3)
O1—Mo1—N2—C15	−55.5 (2)	C26—C13—C14—N2	−63.5 (3)
O2—Mo1—N2—C14	−142.3 (2)	C27—C13—C14—N2	176.8 (3)
O2—Mo1—N2—C15	31.3 (2)	N2—C15—C16—C17	163.0 (3)
O4—Mo1—N2—C14	−40.9 (2)	N2—C15—C16—C25	−23.8 (4)
O4—Mo1—N2—C15	132.6 (2)	C15—C16—C17—C18	−14.9 (5)
N1—Mo1—N2—C14	50.5 (2)	C15—C16—C17—C22	168.7 (3)
N1—Mo1—N2—C15	−136.0 (2)	C25—C16—C17—C18	171.9 (3)
Mo1—O1—C1—C2	−147.8 (2)	C25—C16—C17—C22	−4.6 (4)
Mo1—O1—C1—C10	34.8 (3)	C15—C16—C25—O2	12.1 (4)
Mo1—O2—C25—C16	35.8 (4)	C15—C16—C25—C24	−165.6 (3)
Mo1—O2—C25—C24	−146.5 (2)	C17—C16—C25—O2	−174.6 (3)
Mo1—N1—C11—C10	−11.9 (4)	C17—C16—C25—C24	7.7 (4)
C12—N1—C11—C10	163.0 (3)	C16—C17—C18—C19	−176.5 (3)
Mo1—N1—C12—C13	75.1 (3)	C22—C17—C18—C19	0.1 (5)
C11—N1—C12—C13	−100.2 (3)	C16—C17—C22—C21	176.7 (3)
Mo1—N2—C14—C13	−61.8 (3)	C16—C17—C22—C23	−1.1 (5)
C15—N2—C14—C13	124.3 (3)	C18—C17—C22—C21	0.1 (5)
Mo1—N2—C15—C16	−5.7 (4)	C18—C17—C22—C23	−177.8 (3)
C14—N2—C15—C16	167.9 (3)	C17—C18—C19—C20	0.3 (6)
O1—C1—C2—C3	178.1 (3)	C18—C19—C20—C21	−0.8 (6)
C10—C1—C2—C3	−4.4 (4)	C19—C20—C21—C22	0.9 (5)
O1—C1—C10—C9	−179.4 (2)	C20—C21—C22—C17	−0.6 (5)
O1—C1—C10—C11	12.0 (4)	C20—C21—C22—C23	177.3 (3)
C2—C1—C10—C9	3.1 (4)	C17—C22—C23—C24	3.9 (5)
C2—C1—C10—C11	−165.4 (2)	C21—C22—C23—C24	−174.0 (3)
C1—C2—C3—C4	2.2 (5)	C22—C23—C24—C25	−0.8 (5)
C2—C3—C4—C5	−177.8 (3)	C23—C24—C25—O2	177.1 (3)
C2—C3—C4—C9	1.2 (5)	C23—C24—C25—C16	−5.1 (5)
C3—C4—C5—C6	179.3 (3)		

### Hydrogen-bond geometry (Å, °)

**Table:**

*D*—H···*A*	*D*—H	H···*A*	*D*···*A*	*D*—H···*A*
C6—H6···O3^i^	0.95	2.43	3.328 (5)	159
C11—H11···O4^ii^	0.95	2.38	3.297 (3)	161
C12—H12A···O4	0.99	2.47	2.974 (4)	111
C27—H27A···O3^ii^	0.98	2.55	3.490 (4)	161
C28—H28A···O2^iii^	0.99	2.40	3.213 (5)	139
C28—H28A···O4^iii^	0.99	2.53	3.429 (6)	151

Symmetry codes: (i) −*x*+1/2, −*y*+3/2, −*z*; (ii) −*x*+1/2, *y*−1/2, −*z*+1/2; (iii) *x*, *y*−1, *z*.
